# Molecular Typing and Drug Resistance Analysis of Carbapenem-resistant Klebsiella Pneumoniae from ICU Patients in China

**DOI:** 10.31661/gmj.v13i.3302

**Published:** 2024-01-29

**Authors:** Mengwei Ma, Xian Zhang, Yingli Li, Jingfu Qiu, Jian Xue

**Affiliations:** ^1^ College of Public Health, Chongqing Medical University, Chongqing, China; ^2^ Department of Hospital Infection, The Third Affiliated Hospital of Zunyi Medical University, The First People’s Hospital of Zunyi, Guizhou, China; ^3^ Department of Health Management, Zunyi Medical and Pharmaceutical College, Guizhou, China

**Keywords:** ICU, Carbapenem-resistant Klebsiella Pneumoniae, Molecular Epidemiology

## Abstract

Background: Carbapenem-resistant Klebsiella pneumoniae (CRKP) stands out as one of the most detrimental nosocomial pathogens in Chinese hospitals. The resistance rate of CRKP to carbapenems has persistently remained elevated, particularly in intensive care unit (ICU). This study focused on the molecular epidemiological characteristics of CRKP isolated from Chinese ICU patients.Materials and Methods: Five distinct CRKP isolates were obtained from a Chinese hospital. Strain identification and drug susceptibility testing were conducted using the VITEK® 2 Compact Bacterial Identification and Monitoring System. Whole genome sequencing (WGS) technology was used to analyze sequence typing, phylogenetic relationships and drug resistance genes.Results: All five CRKP isolates carried the carbapenem-resistance gene blaKPC-2 and exhibited complete resistance to β-lactams, aminoglycosides, quinolones, and partial resistance to sulfonamides. Based on the single nucleotide polymorphism differences, we classified the five CRKP isolates into 3 distinct clusters. Multilocus sequence typing (MLST) and core genome multilocus sequence typing (cgMLST) identified the main prevalent sequence type of CRKP as ST11-CT1313.Conclusions: Utilizing WGS for sequence typing, phylogenetic analysis, and antibiotic resistance gene identification is essential in enhancing the control and containment of CRKP infections in ICU. However, it is vital to consider both resistance phenotypes and resistance genes when guiding clinical medication decisions.

## Introduction

Since the initial detection of carbapenem-resistant Klebsiella pneumoniae (CRKP) isolates in 1997 in USA [1], CRKP infections and outbreaks have appeared all over the world [2][3][4]. Owing to its heightened pathogenicity and limited treatment options, patients infected with CRKP experience significantly higher mortality rates compared to those infected with carbapenem-susceptible K. pneumoniae (CSKP) [5][6]. CRKP poses a serious threat to global public health. In 2017, WHO released a list of 12 drug-resistant bacteria for which the development of new antibiotics is considered urgent. Among them, CRKP was categorized as an urgent threat level [7].

The most common cause of carbapenem resistance is the production of carbapenemase [8]. Various genes encoding carbapenemases (blaKPC, blaNDM, blaOXA-48, or blaVIM) have the potential to be horizontally transferred via plasmids, thereby accelerating the conversion of CSKP to CRKP [9]. A nationwide surveillance study conducted from 2016 to 2020 revealed significant changes in the genetic characteristics of Chinese CRKP isolates, which were similar to those of hypervirulent K. pneumoniae (HvKP). Therefore, there is currently a large number of hypervirulent CRKP in China [10]. Notably, K. pneumoniae colonization serves as a critical step in the progression to extraintestinal infections [11].

Considering the easy diffusion, hypervirulence and considerable concealment of CRKP, continuous and efficient surveillance is important for nosocomial infection prevention and control.

Although researchers have tackled the antibiotic resistance crisis by using colistin, antimicrobial peptides and nanoparticles alone or in combination, the problem of antibiotic resistance remains serious [12][13][14]. Before COVID-19 caused a global pandemic, overuse of antibiotics for bacterial infections had already led to widespread antibiotic resistance, making antibiotic resistance a major global public health challenge that is projected to cause 10 million premature deaths per year by 2050 and a global economic loss of 100 trillion USD [15]. Antibiotic resistance has been further aggravated by excessive and incorrect use of antibiotics during the COVID-19 pandemic [16][17]. Therefore, by monitoring and controlling the spread of resistance genes is important to prevent resistance to antibiotics as the last line of treatment.

With the advancements in sequencing technology and the reduction in costs, whole genome sequencing (WGS) has become a powerful tool in nosocomial infection surveillance, enabling precise identification of pathogen sources and transmission routes within hospitals [18]. Previous studies, including our own and those from other research groups, have confirmed the efficacy of WGS in accurately determining sequence typing, phylogenetic relationships and drug resistance genes [19][20].

The Intensive Care Unit (ICU) is a crucial component of modern hospitals, but it is also associated with a high incidence of hospital-acquired infections (HAI) [13]. During the past 10 years, the incidence of HAI in the ICU of general hospitals in China was as high as 26.07%, much higher than that of surgery (3.26%) and internal medicine (3.06%), which followed closely behind [21]. The occurrence of ICU nosocomial infections is inseparable from bacterial drug resistance; the emergence of drug-resistant bacteria contributes to their colonization and reproduction in the human body and peripheral environment, especially those that are partially or fully resistant to existing antimicrobial drugs [7][22]. This study focused on the molecular epidemiological characteristics of CRKP isolated from Chinese ICU patients.

## Materials and Methods

Bacterial Isolates

Five different clinical isolates of CRKP were obtained from a hospital in China. These isolates originated from the ICU of the hospital and the samples were collected between February 2020 and February 2021.

Antimicrobial Susceptibility Testing

All clinical isolates were first screened by the VITEK® 2 Compact Bacterial Identification and Monitoring System, enabling rapid and accurate identification of these isolates. The control strain was Escherichia coli ATCC 25922. The judgement was based on the 2022 standard of the American Clinical and Laboratory Standards Institute (CLSI) [23].

Extraction of Bacterial Genomic DNA.

CRKP bacteria were cultured overnight and centrifuged to collect the bacterial precipitate, then the genomic DNA was extracted using a bacterial DNA kit. Finally, the extraction results were verified by agarose gel electrophoresis.

Whole Genome Sequencing

The WGS of 5 CRKP strains was completed by the Shanghai Sangon Biological Company. The sequencing platform was Illumina HiSeq 2500.

Sequence Typing

Sequence typing was performed using both core genome multilocus sequence typing (cgMLST) and multilocus sequence typing (MLST). The cgMLST used SeqSphere+ software to assign the complex types (CTs), and MLST used the PubMLST database for identification.

Cluster Dendrogram Construction

Cluster dendrograms was generated based on the core genome single nucleotide polymorphisms (cgSNPs) differences between strains using the UPGMA clustering method via R software (version 4.2.0; R Foundation for Statistical Computing, Vienna, Austria). cgSNPs were obtained by comparing the genomes of five CRKP isolates with K. pneumoniae MGH78578.

Drug Resistance Gene Analysis

The sequenced CRKP gene sequences were compared to similar sequences in the reference database, Comprehensive Antibiotic Resistance Database (CARD), using the BLAST software (https://blast.ncbi.nlm.nih.gov/Blast.cgi) from NCBI to determine the resistance gene.

## Results

**Figure-1:**
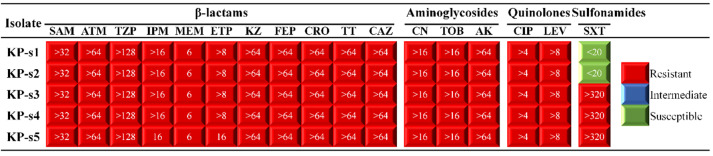
Figure 1. The antimicrobial susceptibility of 5 CRKPs.

**Figure-2:**
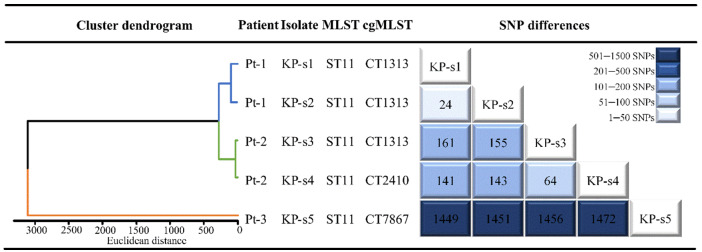
Figure 2. The cluster dendrogram, sequence types and SNP differences of CRKPs based on WGS.

Isolates

Among the 5 CRKP isolates, 2 strains were isolated from sputum (2/5, 40%), 2 strains were isolated from urine (2/5, 40%) and 1 strain was isolated from a fiberoptic bronchoscopic lavage solution (1/5, 20%).

CRKP Susceptibility

The 5 CRKP isolates were completely resistant to β-lactam antibiotics, including ampicillin/sulbactam (SAM), aztreonam (ATM), piperacillin/tazobactam (TZP), imipenem (IPM), meropenem (MEM), ertapenem (ETP), cefazolin (KZ), cefepime (FEP), ceftriaxone (CRO), cefotetan (TT) and ceftazidime (CAZ). They were also completely resistant to aminoglycoside antibiotics, including gentamicin (CN), tobramycin (TOB) and amikacin (AK); completely resistant to quinolone antibiotics, including ciprofloxacin (CIP) and levofloxacin (LEV); and partially resistant to the sulfonamide antibiotic sulfamethoxa-zole/trimethoprim (SXT) (3/5, 60%), as shown in Figure-1.

CRKP Sequence Typing

The sequence types (ST) of all 5 CRKP isolates identified by MLST was ST11. The cgMLST revealed three CTs, including one new CT (CT7867). The 3 distinct CTs, including the 3 strains belonging to CT1313 (3/5, 60%), 1 strain to CT2410 (1/5, 20%) and 1 strain to CT7867 (1/5, 20%), are shown in Figure-2.

CRKP Cluster Dendrogram

As depicted in Figure-2, a cluster dendrogram was constructed based on the cgSNPs. A careful examination of the different cgSNPs among the 5 strains revealed notable variations. d-s5, isolated from patient 3 (Pt-3), exhibited the largest single nucleotide polymorphisms (SNPs) difference compared to the other strains (1400-1500 SNPs). KP-s1, isolated from patient 1 (Pt-1), had the smallest SNP difference from KP-s2 (24 SNPs), followed by KP-s3 and KP-s4, isolated from patient 2 (Pt-2) (64 SNPs). Based on the SNP differences, the 5 CRKP isolates were clustered into 3 distinct groups. Cluster 1 comprised 2 strains (KP-s1 and KP-s2) isolated from Pt-1; cluster 2 consisted of 2 strains (KP-s3 and KP-s4) isolated from Pt-2; and cluster 3 included 1 strain (KP-s5) isolated from Pt-3.

Characterization of Resistance Genes

**Figure-3:**
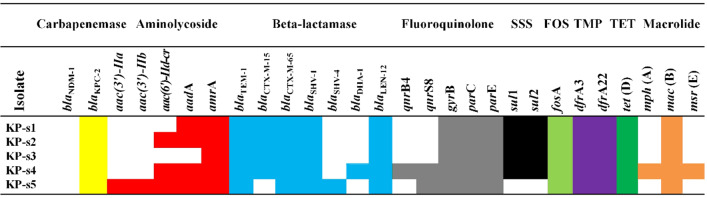
Figure 3. The characterization of resistance genes among the CRKPs

As shown in Figure-3, they carried the carbapenem resistance gene blaKPC-2 (5/5, 100%), aminoglycoside resistance genes aac(3’)-Ⅱa (1/5, 20%), aac(3’)-Ⅱb (1/5, 20%), aac(6’)-Ⅱd-cr (3/5, 60%), aadA (4/5, 80%) and amrA (5/5, 100%), β-lactam resistance genes blaTEM-1 (5/5, 100%), blaCTX-M-15 (4/5, 80%), blaCTX-M-65 (5/5, 100%), blaSHV-1 (5/5, 100%), blaSHV-4 (1/5, 20%), blaDHA-1 (1/5, 20%) and blaLEN-12 (5/5, 100%), fluoroquinolone resistance genes qnrB4 (1/5, 20%), qnrS8 (2/5, 40%), gyrB (5/5, 100%), parC (5/5, 100%) and parE (5/5, 100%), sulfonamide (SSS) resistance genes sul1 (4/5, 80%) and sul2 (4/5, 80%), fosfomycin (FOS) resistance gene fosA (5/5, 100%), trimethoprim (TMP) resistance genes dfrA3 (5/5, 100%) and dfrA22 (5/5, 100%), tetracycline (TET) resistance gene tet (D) (5/5, 100%), macrolide resistance genes mph (A) (1/5, 20%), mac (B) (5/5, 100%) and msr (E) (1/5, 20%).

## Discussion

CRKP represents a grave nosocomial threat in Chinese hospitals, particularly within the ICU setting [24][25]. Studies have shown that ICU stays increase the risk of CRKP infections and raise the mortality risk in infected patients [26]. CRKP has consistently exhibited a severe level of resistance to carbapenem antibiotics. For instance, CHINET surveillance data from 2005 to 2021 demonstrated a rapid increase in resistance rates of K. pneumoniae to IMP and MEM, reaching 25.0% and 26.3% in 2018, respectively, and maintaining a detection rate of over 23% from 2019 to 2021 [27].

Currently, resistance and virulence-associated gene detection and clonal relationship analyses are mainly performed by PCR, MLST and PFGE methods in the clinical setting [28]. However, these methods are complex and have limited resolution, especially when dealing with highly similar clones, making it difficult to distinguish them. On the other hand, the integration of WGS and cgMLST offers a higher discriminatory power. It can analyze a thousand SNPs sites in the core genome to accurately classify subclone and tracking outbreaks [29][30].

In this study, based on the WGS technique, the main prevalent sequence type of CRKP classified by MLST in the ICU of this hospital was ST11, aligning with findings reported in other ICUs in China [31][32]. After accurate typing by cgMLST, the five CRKP strains of ST11 could be further divided into three CTs; the main one was CT1313, which was consistent with other reports from ICU in China [29]. By integrating the clinical data and analyzing the phylogenetic tree, the five CRKP strains were found to be classified into three distinct clusters, with each cluster associated with different patients.

Drug resistance testing revealed that all five CRKP strains exhibited complete resistance to β-lactams, aminoglycosides and quinolones. They were also partially resistant to sulfonamides. Analysis of resistance genes revealed that all five CRKP strains carried the gene blaKPC-2, suggesting that KPC-2 carbapenemase production was the primary resistance mechanism responsible for carbapenem resistance in the ICU of this hospital, consistent with reports from other Chinese ICUs [31][33]. The primary mechanism leading to SXT resistance has been reported to be the high prevalence of resistance genes, including the sul and dfr genes [34]. However, these resistance genes did not always align with the phenotypic resistance results. Our results showed that KP-s1 and KP-s2, although carrying the sul gene, were sensitive to SXT, which was consistent with previous studies [35][20]. EUCAST also noted that for most bacteria, guiding the clinical use of antibiotics should not depend solely on the type of resistance genes carried by the bacteria [36]. Therefore, it is necessary to combine the resistance phenotype and resistance genes when guiding clinical medication.

As a special group, ICU patients are in a critical condition and are exposed to a complex environment, receiving frequent invasive operations and large amounts of antibiotics. Extensive research has demonstrated that an extended duration of hospitalization, prior utilization of carbapenems, invasive interventions, employment of intravascular catheters, and tracheotomies serve as risk factors for contracting CRKP infections [26][37]. Consequently, the prompt implementation of WGS enables the accurate acquisition of patients’ etiological information, thereby expediting the administration of antibiotics, reducing the variety of antibiotic types, and facilitating precise targeted treatment.

## Conclusion

The clustering dendrogram of the five CRKP strains isolated from an ICU based on the WGS technique could be classified into three clusters consistent with their sample sources, implying the accuracy of the WGS classification. cgMLST based on WGS technology was more discriminative than MLST and the five CRKP strains of ST11 could be further classified into three CTs. Furthermore, all five CRKP isolates harbored the carbapenem-resistance gene blaKPC-2, along with several other resistance genes. However, bacteria may have multiple resistance mechanisms, so relying on resistance gene types alone may not adequately capture the complete bacterial response to antibiotics. Both the resistance phenotypes and resistance genes need to be combined when guiding clinical medication.

## Acknowledgement

This research was funded by Guizhou Provincial Health Commission [2024GZWJKJXM1416].

## Conflict of Interest

The authors declare that they have no conflict of interest.
